# Comparative Overview of the Performance of Cementitious and Non-Cementitious Nanomaterials in Mortar at Normal and Elevated Temperatures

**DOI:** 10.3390/nano11040911

**Published:** 2021-04-02

**Authors:** M. Arsalan Khan, M. Khalid Imam, Kashif Irshad, Hafiz Muhammad Ali, Mohd Abul Hasan, Saiful Islam

**Affiliations:** 1 Department of Civil Engineering, Z. H. College of Engineering and Technology, Aligarh Muslim University, Aligarh 202002, India; mohd.arsalan.khan@hotmail.co.uk; 2 Center of Research Excellence in Renewable Energy, King Fahd University of Petroleum & Minerals, Dhahran 31261, Saudi Arabia; Kashif.irshad@kfupm.edu.sa; 3 Mechanical Engineering Department, King Fahd University of Petroleum and Minerals, Dhahran 31261, Saudi Arabia; hafiz.ali@kfupm.edu.sa; 4 Civil Engineering Department, College of Engineering, King Khalid University, Abha 61421, Saudi Arabia; mohad@kku.edu.sa (M.A.H.); sfakrul@kku.edu.sa (S.I.)

**Keywords:** nano-mortar, fresh-state properties, mechanical properties, durability properties, microstructural characterization, elevated temperature

## Abstract

Nanotechnology has emerged as a field with promising applications in building materials. Nanotechnology-based mortars are examples of such building materials that have widespread applications in the construction industry. The main nanomaterials used in mortars include nano-silica, nano-magnesium oxide, nano-alumina, nano-titanium oxide, nano-zinc oxide, nano-clay, and nano-carbon. This review paper presents a summary of the properties and effects of these nanomaterials on cement mortar in terms of its fresh-state and hard-state properties. The fresh-state properties include the setting time, consistency, and workability, while the hard-state properties include mechanical properties such as compressive, flexural, tensile strengths, as well as the elasticity modulus, in addition to durability properties such as water absorption, shrinkage strain, strength loss due to freeze–thaw cycles, and chloride penetration, among others. Different nanomaterials cause different physical and chemical alterations within the microstructures of cement mortar. Therefore, the microstructural characterization and densification of mortar are discussed in detail at varying temperatures. In general, the involvement of nanomaterials in cement mortar influences the fresh-state properties, enhances the mechanical properties, and impacts the durability properties, while reducing the porosity present in the mortar matrix. Cementitious nanomaterials can create a pathway for the easy injection of binding materials into the internal microstructures of a hydration gel to impact the hydration process at different rates, whereas their non-cementitious counterparts can act as fillers. Furthermore, the research gaps and future outlook regarding the application of nanomaterials in mortar are discussed.

## 1. Introduction

In nanotechnology-based mortar, cement is the second most consumed material after water and the main constituents. In generally, the word cement refers to any binder that sets, hardens, and firmly holds other materials. The most widely used cements are inorganic and hydraulic, including Portland cement and other cements (for example, calcium aluminate cement, supersulfated cement, calcium sulfoaluminate cement, and geopolymer cement). Cement is rarely used on its own, but it is used with sand to produce mortar or with sand and gravel to produce concrete. Therefore, cementitious composites refer to all materials made from cement as a generalized concept [1]. However, cement has faced much criticism for causing serious environmental problems, particularly greenhouse effects, due to the release of large amounts of carbon dioxide during its production [2]. Nasr et al. [3] suggested that cement powder could be partially replaced with other cementitious materials (without compromising the quality of mortar) to allow sustainable development in the construction industry. In this regard, Pozzolan is defined as a siliceous or siliceous and aluminous material, which in itself possesses little or no cementitious activity, but in a finely divided form—and in the presence of moisture—will chemically react with calcium hydroxide at ordinary temperatures to form compounds possessing cementitious properties. Pozzolanic materials are added to ordinary Portland cement to produce Portland pozzolan cement. Pozzolanic materials help in reducing OPC production, which in turn reduces the amount of carbon dioxide released into the atmosphere [4]. Synthetic pozzolanic materials (such as ground, granulated blast furnace slag (GGBFS), micro-silica, fly ash, and others) have been widely used to improve the durability and mechanical properties of mortar [5]. However, with the advancements of science and technology and the development of nanotechnology, nanomaterials such as nano-silica, nano-carbon, nano-titanium oxide, nano-clay, and nano-alumina have been used to improve mortar properties; as the well-known Richard Feynman (1959) rightly stated, “There is plenty of room at the bottom [6]”.

Researchers have shown extensive interest in the synthesis and use of nanomaterials in the last few decades [7], including their great contributions towards construction and building engineering. This has encouraged studies on the structure of the mortar matrix at the nano-level and on properties related to changes at the atomic or molecular scale. In several studies, nanomaterials have been used along with other mineral admixtures, such as zeolite, blast furnace slag, silica fume, fly ash, and rice husk ash [3,8,9,10,11]. Nanomaterials are also used with fibers, such as hemp and polyvinyl alcohol fibers [12,13]. The inclusion of nanofillers such as nano-silica, nano-TiO_2_, and nano-ZrO_2_ in cement-based compounds is due to their small size and ability to fill spaces, which improve the compactness. The pozzolanic effects of nanofillers enhance the strength of cementitious compounds, while the nucleation effect and the core effect of nanofillers hinder the propagation of cracks, helping to improve the weak interfaces of cementitious compounds [14]. The use of nanomaterials, especially nano-silica, in concrete is an emerging innovative approach that may have the potential to extend the service life of reinforced concrete infrastructure. Recently, several researchers concluded that the use of nanomaterials in concrete is effective in order to improve the mechanical properties and durability. However, very few studies are available on the inclusion of nanomaterials in mortar, mainly due to the fact that is not considered a structural building block. However, mortar serves as a building element in masonry structures and as an envelope when used as plaster. Our aim is to address this research gap by investigating the roles of nanomaterials in mortar in terms of its ability to affect the durability and thermal and practicality aspects. This will also help in providing comprehensive recommendations for the utilization of nanomaterials in day-to-day construction practices, which are still awaited.

The effects of the nanomaterials on mortar properties, such as the workability, compressive and flexural strengths, water absorption, chloride penetration, and others, are examined through experimental studies [15]. The sonication process is used for dispersion of nanomaterials along with surfactants to overcome the drawbacks of the nanomaterials, which show improper dispersion during mixing with mortar ingredients [16]. Dong et al. [17] recommended the use of reinforced, nickel-plated carbon nanotubes in cementitious compounds to achieve high dispersion, which helps to improve effects on the mechanical properties at low dosages. Depending on the chemical reactivity, surface area, and other properties, the nanomaterials can remarkably affect the durability and mechanical properties of cement mortar [18]. For example, pertaining to their large surface area and tiny size, nanomaterials accelerate the cement hydration process and pozzolanic reactions [19].

The mechanical properties, such as the flexural, tensile, compressive, and shear strengths, as well as the elasticity modulus, are enhanced with the inclusion of nano-silica [20]. The mechanical impact properties are improved by the incorporation of nanofillers. Cementitious compounds with nano-silica have the highest dynamic maximum strain, while compounds containing nano-ZrO_2_ and nano-TiO_2_ have the maximum dynamic strain and dynamic compressive strength [14]. The involvement of nanomaterials also impact durability properties, such as resistance to water absorption, shrinkage strain, chloride resistance, etc. [21]. In [22], it was observed that incorporation of nano-silica, nano-titanium, or nano-ZrO_2_ in other cementitious compounds such as reactive powder concrete (RPC) improved the wear resistance and resistance to chloride penetration with modification of the microstructures. Cementitious nanomaterials directly impact the hydration process. It was observed that the pozzolan nanofillers accelerate the progression of the hydration of the cementitious compounds. The inclusion of nanofillers in cementitious compounds improves the rigidity and hardness of the calcium silicate hydrate (C–S–H) gel by decreasing the orientation of the CH crystals, reducing the size of the CH in the case of pozzolan nanofillers, and by increasing the amount of CH at early age, modifying the orientation index of the CH crystals in the case of a non-pozzolanic nanofiller [23]. The mechanisms of the effects of nanofillers in C–S–H gel can be attributed to the nucleation effect and pozzolanic effect (for nano-silica) of nanofillers, which facilitate hydration of cement and the ability for high water absorption in nanofillers, reducing the amount of proton water in the C–S–H gel and shortening the distance between the structural groups of the Ca, O, and Si atoms [24]. The interfacial microstructures of the nanocomposites are more compact and the content and size of the calcium hydroxide crystals are significantly reduced compared to compounds without nanofillers [25]. The experimental results show that the incorporation of all types of nanofillers reduces the porosity of the cementitious compounds by converting the water from the pores of the C–S–H gel, which induces a reorganization of the structure of the gel and causes shrinkage of gel pores and fine capillary pores [26].

Even though various aspects of the effects of nanomaterials on cement mortar have been explicitly studied recently, the literature does not provide a holistic view that includes the behaviour at elevated temperatures. This paper is an attempt at a thorough and exhaustive review of the mechanical and durability properties, as well as microstructural characterization. The mechanical properties include the compressive, tensile, and flexural strengths and the elasticity modulus, while the durability properties include chloride penetration, water absorption, strength loss due to freeze–thaw cycles, shrinkage strain, and other factors. All of the major effects are carefully presented and demonstrated within by covering a wide collection of research papers on cement mortars with the inclusion of nanomaterials, leading to recommendations for future research.

## 2. Materials 

**Figure 1:**
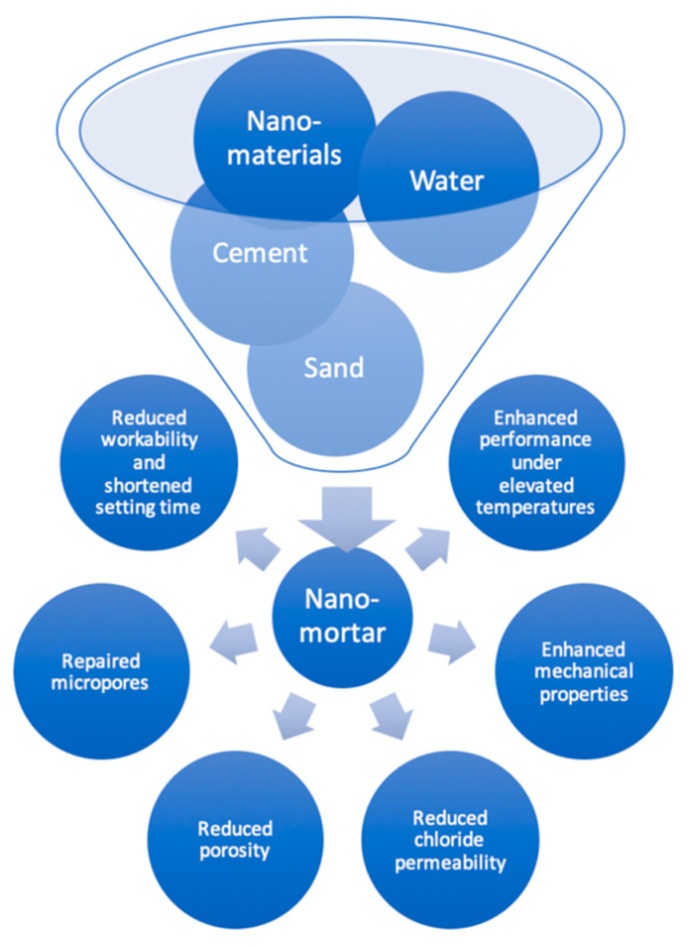
Constituents of nanomaterials added to mortar and outcomes.

The cement, sand, nanomaterial(s), and water form important constituents of mortars incorporating nanomaterials (Figure 1).

Sometimes, the properties of a mortar are also enhanced by adding superplasticizers and admixtures. The properties of nanomaterials are represented in Table 1, while the remaining pozzolan constituents of cementitious composites are elaborated in Table 2.

### 2.1. Cement

In experimental studies, ordinary Portland cement (OPC) at strengths of 42.5 or 52.5 MPa according to ASTM specifications is often used in cement mortar [69,70,71,72]. Generally, OPC Type I, Grade 42.5 N is used, conforming to ASTM C-150 [73,74,75,76]. Some researchers have used OPC Type II, conforming to ASTM C-150 [77], while in a few studies OPC has been partially replaced by weight with Class F fly ash or rice husk ash [30,78].

### 2.2. Sand

Sand acts as a fine aggregate and is usually made from locally available natural silicate sand, with maximum nominal sizes between 4.75 and 1.18 mm [29,79]. Sometimes, commercial sand, mining sand, natural river sand, Indian standard sand (Ennore sand), and quartz sand are used, with maximum sizes of 1.2, 2.36, 1.18, 2, and 2 mm, respectively [8,80,81,82,83]. The fineness moduli of the fine aggregates used in various experimental studies in mortar vary between 2.4 and 2.8, while the specific gravity values vary between 2.45 and 2.65 [29,73,84,85]. The binder/sand ratio is generally kept between 1:2 and 1:4 [36,78,86,87]. ASTM C-778 is used to identify the grading curve of a fine aggregate [84].

### 2.3. Nanomaterials

Nano-silica or nano-SiO_2_ is used in three forms: powdered form, sol form, and gel type, and is the most widely used nanomaterial, not only because of its benefits, but also due to relatively low cost [88]. Nano-silica occurs in nature in the form of quartz. Acid leaching and milling processes are then used to convert it into powdered silica nanoparticles [89]. This powdered form of nano-silica contains amorphous crystals with spherical morphology [60]. Polymerization of silicic acid in the aqueous medium is used to process the nano-silica sol form. The sol–gel technique is used to produce nano-silica gel by destabilization of the nano-silica sol and then consequent drying. Nano-silica impacts the strength and durability of cement mortar, not only due to physical alterations, but mainly chemical reactions. The most important advantage of nano-silica is in the formation of calcium silicate hydrate (C–S–H), as represented by Equation (1).

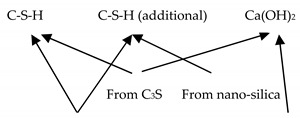
Cement + H_2_O + nano-silica → Ca^2+^ + H_2_SiO_4_^2−^ + H_2_SiO_4_^2−^ + OH^−^             Nano-SiO_2_ + H_2_O → H_2_SiO_4_^2−^             Ca(OH)_2_ + H_2_O → Ca^2+^ + OH^−^                    H_2_SiO_4_^2−^ + Ca^2+^ → C–S–H (additional C–S–H)             C_3_S + H_2_O → C–S–H + Ca(OH)_2_              *C–S–H = calcium silicate hydrate(1)

Nano-clay (nano-CaCO_3_) acts as a promoter for cement hydration processes. The reasons for this is that nano-clay (Nano-CaCO_3_) makes the diffused Ca^2+^ accumulate on its particle surfaces, decreasing the nearby Ca^2+^ concentration, and thus accelerating the chemical reaction of C_3_S [90]. Depending on the chemical morphology and composition of the crystalline, it can be of montmorillonite, kaolinite, halloysite, illite, or vermiculite type [91].

Nano-alumina, in powdered form with flake morphology, has very good applications as a building material and is utilized as a partial substitution for binders in cement composites. The dissolution process is demonstrated in Equation (2). Dissolved nano-alumina precipitates as monosulfate. The formation of monosulfate and monocarbonate related to the presence of nano-alumina is demonstrated in Equations (3) and (4), respectively, which leads to a decrease in porosity, and therefore leads to an improvement in strength by increasing the formation of solids [45].

Al_2_O_3_·xH_2_O + 2OH^−^ → 2Al(OH)_4_^−^ + (x − 3)H_2_O (2)

2Al(OH)_4_^−^ + 3Ca(OH)_2_ + Ca^2+^ + SO_4_^2−^ + 6H_2_O → 3CaO·Al_2_O_3_·CaSO_4_·12H_2_O + 2OH^−^ (3)

2Al(OH)_4_^−^ + 3Ca(OH)_2_ + Ca^2+^ + CO_3_^2−^ + 5H_2_O → 3CaO·Al_2_O_3_·CaCO_3_·11H_2_O + 2OH^−^ (4)

Nano-magnesium oxide is used as an additive for partial replacement of cement and has a particle size of less than 420 nm, having specific surface area (SSA) of 25 to 50 m^2^/g. The nano-MgO particles affected the microstructure of the hardened pastes and had a denser matrix compared to simple composites due to the filling and expansion effects of Mg(OH)_2_, possibly another cause of modification of the mechanical properties of mortars. The use of MgO-based expansive additives for the production of cement-based cohesive products, such as magnesium silicate hydrates at later ages, can complement the formation of C–S–H products at early ages and contribute to further intensification of the microstructure [92].

Nano-zinc oxide is another nanomaterial that is used in powdered form to improve the durability and mechanical properties of cement mortar. Replacing cement with nano-zinc oxide appears to increase the structure of the hydrated calcium silicate, which leads to improved hydration of the cement paste [54].

Nano-titanium oxide is a white pigment material with a spherical shape, which is used as partial replacement for binder due to its high specific surface area [93]. For extraction at the industrial level, the sol–gel method is widely used on nano-titanium oxide [94].

Nano-carbon materials include carbon nano-fibers (CNFs) and carbon nano-tubes (CNTs) [95]. CNTs, classified into single-walled CNTs (SWCNTs) and multi-walled CNTs (MWCNTs), are produced by exfoliation of natural graphite powder [73] and are used both as liquid solutions and in powdered form [96]. SWCNTs are graphite tubes with their ends capped, whereas MWCNTs are concentric cylindrical graphite tubes made out of single-walled CNTs. MWCNTs are most widely used, as they are better reinforcing materials and are cost effective [61].

### 2.4. Water and Admixtures

The water used for mixing is either tap water or distilled water [8,43]. The water/binder ratio is generally maintained between 0.3 to 0.5 [42,97,98]. For uniform dispersion of nanomaterials and to give the desired level of workability to the mix, polycarboxylate-ether-based or naphthalene-sulfonate-based superplasticizers are added according to ASTM C-494 Type F [50,99]. Sometimes, to improve the strength, durability, and microstructural characteristics, mineral admixtures such as fly ash or silica fume are used as partial replacements for cement [8,100].

## 3. Properties and Method

From the literature, it is clear that the responses of mortar with the inclusion of cementitious and non-cementitious nanomaterials should be differentiated throughout its preparation cycle, based on its fresh-state and hard-state properties. Depending on the type of nanomaterial, the mortar will undergo various changes, ranging from the hydration process to microstructural alterations. Therefore, the effects of nanomaterials on the fresh-state, mechanical, and durability properties, as well as on the microstructural characterization of mortar and cement paste, are discussed in this section.

### 3.1. Fresh-State Properties

#### 3.1.1. Consistency and Setting Times

**Figure 2:**
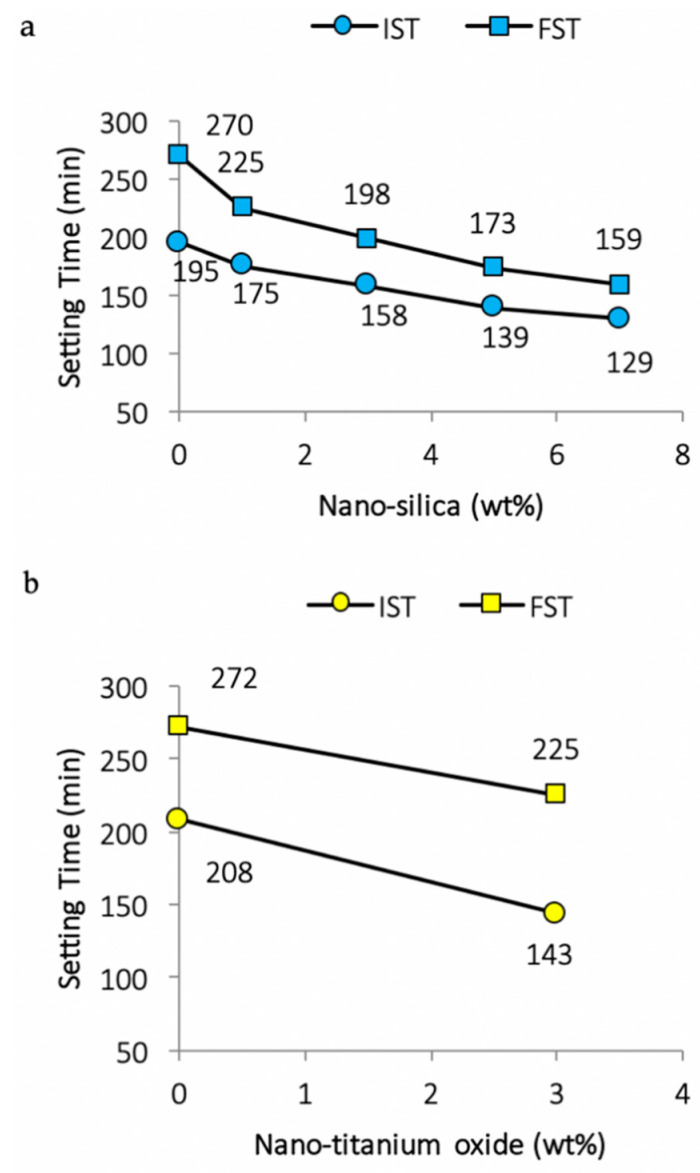
Variations of initial and final setting times due to (**a**) nano-silica [34] or (**b**) nano-titanium oxide in cement paste [102].

A Vicat apparatus as per ASTM C-187 and C-191, respectively, was utilized to measure standard consistency and setting times [35,79]. It is clear from Figure 2 that once proper dispersion of nano-silica is achieved, the setting time accelerates [20,28]. Similarly, the inclusion of nano-TiO_2_, nano-clay, and nano-alumina leads to absorbance of some of the water due to their high surface areas and fineness, which increase the water demands and shorten the initial setting time (IST) and final setting time (FST) [41,57]. For composite cement pastes, which after the addition of ground blast furnace slag (GBFS) and superplasticizer comprise OPC–GBFS–nano-clay, the consistency decreases and setting times increase [101]. The same is true for the addition of a superplasticizer with a nano-clay, which leads to a decrease in water absorption and increases the initial setting times [18]. 

**Figure 3:**
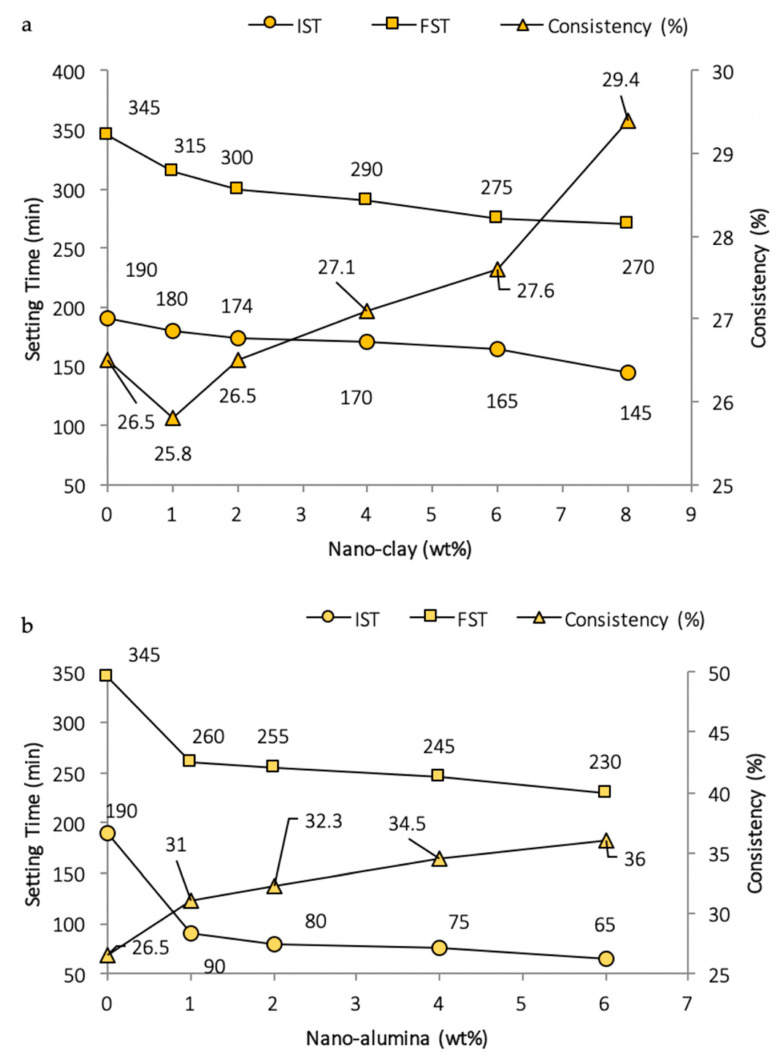
Variations of consistency and setting time due to (**a**) nano-clay [101] or (**b**) nano-alumina in cement paste [46].

The addition of nano-silica increases the water demands with standard consistency and shortens the setting times (refer to Figure 3). This is due to the formation of additional C–S–H particles (summed up in Table 3), which spread in the water-filled spaces between the cement paste and aggregate grains. Thus, it serves as a seed for the production of more compact C–S–H phases [103]. Early solidification and stiffening of the matrix is due to the filling of spaces with the rapid depletion of free mixing water [58,102,104].

#### 3.1.2. Workability (Slump)

**Figure 4:**
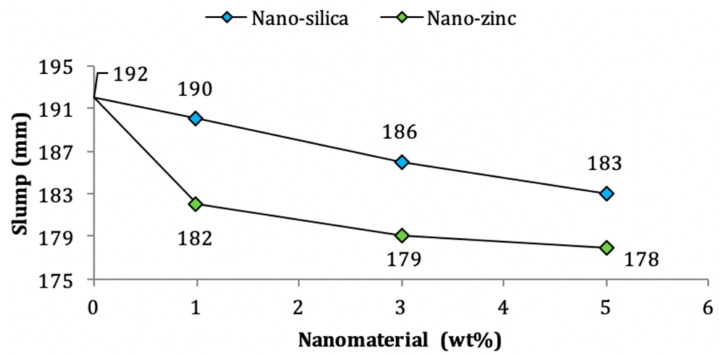
Variations of slump flow due to effects of nano-silica and nano-zinc on cement mortar [55].

A mini slump cone test is used to perform workability tests according to ASTM C-230 [35]. This mini cone has height of 60 mm, bottom and top diameters of 100 mm and 70 mm respectively [81]. Figure 4 indicates that inclusion of nano-silica results in lower slump flow values as compared to control mixes, this may be due to the high surface area of nano-silica which leads to higher absorption of free water [15,30]. 

Likewise, as indicated in Table 4, addition of nano-alumina and nano-zinc reduces the workability of the mortar mix as they have small particle size and more surface area [109,119].

### 3.2. Mechanical Properties

#### 3.2.1. Compressive Strength

**Figure 5:**
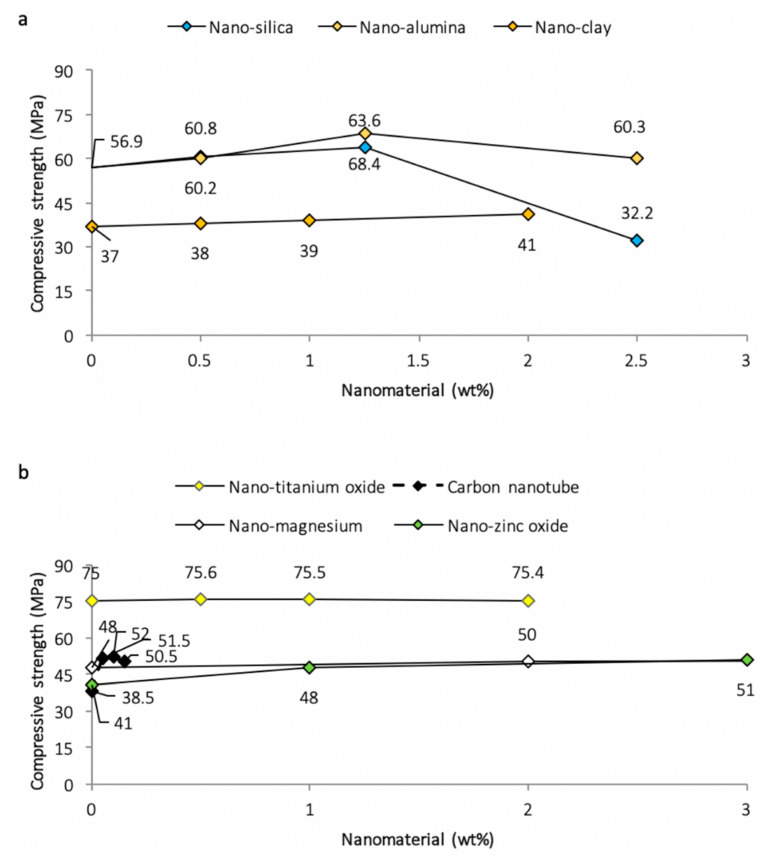
Variations of 28-day compressive strength in cement mortar due to: (**a**) nano-silica and nano-alumina [30] and nano-clay [79]; (**b**) nano-titanium oxide [82], carbon nanotubes [54,110], nano-magnesium oxide [52], and nano-zinc oxide [54].

Following the procedures for ASTM C-109 and ASTM C-39, respectively, the compressive strength for the mortar is determined using cube sizes of 50 or 40 mm and cylinders of 100 mm diameter and 200 mm height for a required number of days [84,123,124,125]. The load is applied using a hydraulic press at a rate of 0.25 MPa/s and an average compressive strength of 3 samples is considered [31,73,87]. The partial replacement of cement with nano-silica (Figure 5a) results in an improvement in compressive strength up to an optimum dosage, beyond which any addition of nano-silica causes a gradual decrease in strength [33,34,83,88,126,127]. This strength enhancement can be attributed to the accelerated cement hydration process, along with the pozzolanic reaction of the nano-silica. A denser and stronger matrix is formed by the nano-silica after reaction with calcium hydroxide to form the C–S–H gel [19,128]. The reduction in strength beyond the optimum dosage is due to uneven dispersion and agglomeration of nano-silica in the matrix, leading to formation of weak zones within the mortar matrix [78].

**Figure 6:**
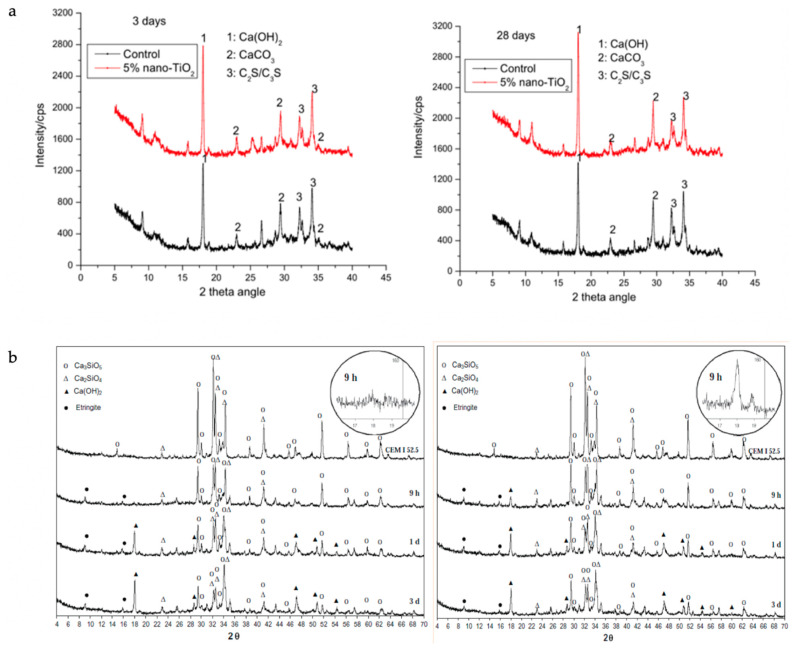
XRD patterns of cement pastes before and after the addition of: (**a**) nano-TiO_2_ (taken with permission from Zhang et al. [21]); (**b**) nano-silica (taken with permission from Sneff et al. [86]).

Similarly, as demonstrated in Figure 5 and the key observations compiled in Table 2, the addition of other nanomaterials such as nano-alumina, nano-MgO, nano-ZnO, nano-TiO_2_, nano-clay, and carbon nanotubes (CNT) up to the optimum dosage improves the compressive strength [52,54,56,61,91,99,129,130,131]. Clearly from the XRD images (Figure 6), the compressive strength increase is due to the flaw-bridging effect and filler effect at the nano level [45,132,133,134,135,136], while agglomeration prevents the hydration process due to the formation of weak bonds within the mortar matrix [44,137,138,139,140,141]. From the XRD images (Figure 6), it is quite clear that the formation of CSH compounds occurs from the start of mixing in the case of nano-silica, while it is neutral in the case of nano–titanium oxide.

Farzadnia [48] explained the effects on heat of hydration and studied the impacts of elevated temperatures (up to 1000 °C) for nano-alumina mortar. As the temperature increased, a significant decay in compressive strength was noticed from 200 °C onwards. This was studied in conjunction with increased mass loss and decreased ultrasonic pulse velocity. A sudden drop in compressive strength was seen at 300 to 1000 °C; this may have been due to degradation of portlandite at around 450 °C, as well as degradation of C–S–H, which starts at round 250 °C and finishes at 450 °C [142]. In other studies, an increase in compressive strength was observed against the incorporation of nano-silica and nano-metakaolin in high strength mortar at 400 and 250 °C, respectively [75,143]; this may be due to the higher rate of hydration due to the higher availability of SiO_2_. In [103], it was also observed that for nano-silica mortar, the compressive strength increased up to 450 °C and then decreased at higher temperatures up to 1000 °C.

#### 3.2.2. Flexural and Tensile Strength

**Figure 7:**
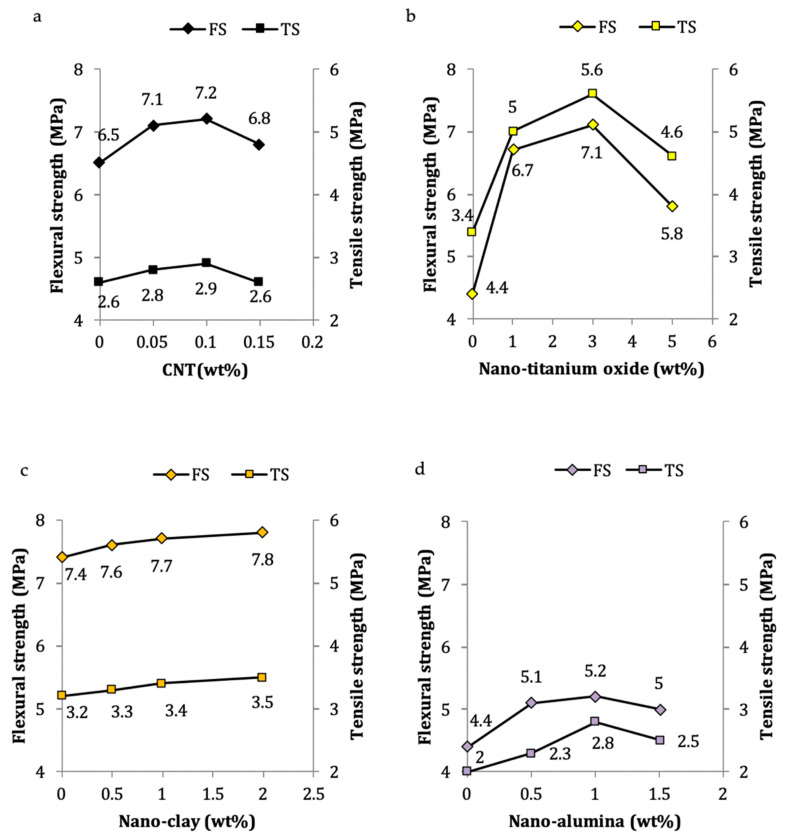
Variations of 28-day flexural and tensile strengths in cement mortar due to: (**a**) CNT [110]; (**b**) nano-titanium oxide [137]; (**c**) nano-clay [79]; (**d**) nano-alumina [113].

The flexural strength (FS) of a mortar is determined by the 3-point bending method, with a displacement rate of 0.5 mm/min on a specimen measuring 40 × 40 × 160 mm^3^ in, accordance with ASTM C-348 [84,124]. The split tensile strength is carried out in accordance with ASTM C-496, using a cylindrical specimen measuring 100 mm in diameter and 200 mm in height [29]. The inclusion of nanomaterials that act as promoters for pozzolanic reactions and increase the production of C–S–H, such as nano-silica and nano-clay, are found to cause a significant overall increase in the flexural strength (FS) and tensile strength (TS) of mortars [13,79,144]. It is also clear from Table 2 that partial replacement of other nanomaterials that densify the C–S–H gel by filling the voids, such as nano-carbon, nano-alumina, nano-titanium, and nano-magnesium oxide with binders, enhance the flexural and tensile strengths [53,57,63,64,73,113,139]. It is important to note form Figure 7 that the FS and TS parameters are observed to follow same pattern corresponding to any specific nanomaterial; among these, the addition of nano-titanium results in a sudden surge in the initial values.

Furthermore, other mechanical properties such as the elasticity modulus and shear strength are also affected with the use of nanomaterials (Table 3). For example, the inclusion of nano-alumina and nano-carbon clogs the pores within the mortar matrix and increases the overall elasticity modulus [48,95]. However, to achieve maximum improvements, the optimum percentages of different nanomaterials may vary. The incorporation of nano-silica impacts the strength and stiffness of the cement mortar; for example, it increases the shear strength of the mortar as it fills the voids in the matrix [32].

### 3.3. Durability Properties

#### 3.3.1. Chloride Penetration 

**Figure 8:**
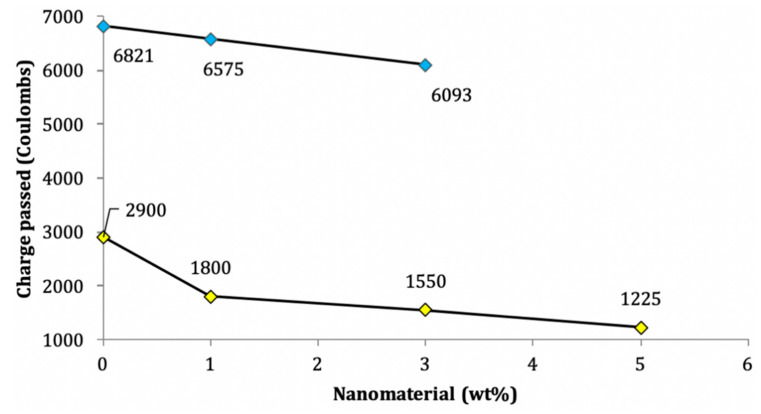
Variations of chloride penetration due to: (**blue diamond**) nano-silica [81]; (**yellow diamond**) nano-titanium oxide on cement mortar [118].

Following ASTM C-1202, the total charge is measured by passing an electric potential of 60 volts for 6 h through the cylindrical specimen measuring 100 mm diameter and 50 mm in height [3]. As summed up in Table 3, the chloride ion penetration of the mortar sample is represented by the total charge in coulombs passing through the mortar specimen. As shown in Figure 8, a decrease in the chloride ion penetration is found with the addition of nano-silica and nano-clay. This is further supported by the works of Zhang and Li [145] and Shaikh and Supit [28]. The increase in the chloride penetration resistance of the cement mortar is attributed to the reduction of hydroxyl groups (OH^−^ ions). The reason for this phenomenon is that nanomaterials with high pozzolanic activity consume calcium hydroxide, which decreases the number of OH^−^ ions [3,120], while the addition of nano-titanium oxide and CNT increases the chloride penetration resistance by decreasing the number of pores that densify the microstructure [10,121].

#### 3.3.2. Water Absorption 

**Figure 9:**
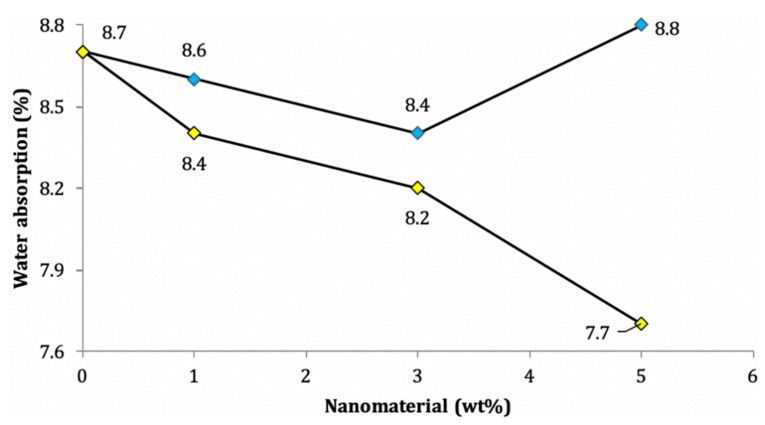
Variations of water absorption in cement mortar due to: (**blue diamond**) nano-silica; (**yellow diamond**) nano-titanium oxide [118].

The guidelines for ASTM C-642 are followed to determine the water absorption (WA). This requires an initial evaluation of the oven-dried mass of the cube specimen (measuring 50 or 40 mm) at the age of 28 or 90 days, or any required number of days. Then, the dry mass values of the specimens are measured after storage at ambient temperature in the laboratory. The saturated surface dry (SSD) mass values are then obtained by saturating the dried specimens in water for 48 h at 21 °C. Next, the SSD mass is divided by the dry mass of the specimen to determine the WA [3]. The addition of pozzolanic nanomaterials such as nano-silica and nano-clay decreases the WA (%) by reducing the permeability; this is because they produce calcium hydroxide to form C–S–H gel, which fills up the pores [3,134]. Other nanomaterials, such as nano-titanium and nano-alumina, act as fillers to densify the interfacial transition zone and the microstructure, which consequently reduces the WA (%) [93,117,146]. Key observations for different nanomaterials are compiled in Table 2. More precisely, Figure 9 shows that nano-titanium (as filler) decreases the WA (%) more rapidly (5.7% per 1 wt% up to 3 wt%) compared to pozzolanic nano-silica (3.4% per 1 wt% up to 3 wt%). After this, the WA requirements increase for nano-silica due to the formation of C–S–H (additional gel).

**Figure 10:**
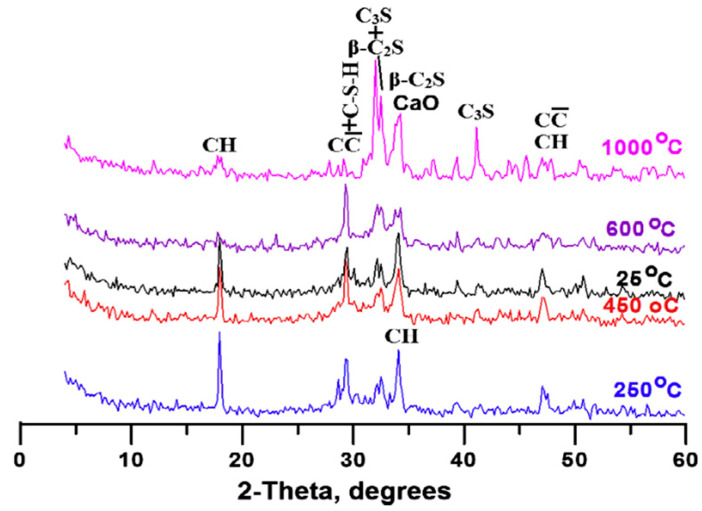
XRD patterns of I.3 cement pastes containing 4 mass% of NS as a function of treatment temperature up to 1000 °C (taken with permission from [103]).

While at elevated temperatures (from 300 °C upwards), [48] indicated increased gas permeability with the addition of nano-alumina in mortar; this was related to loss of mass due to increased evaporation of free water. The high water content in samples was associated with the high surface area of nano-alumina, while opposite behavior was observed in samples without nano-alumina. This may be due to the presence of silica fumes, which accelerate the hydration process. Heikal et al. [103] studied the ignition loss on the inclusion of nano-silica in mortar. Maximal increase in ignition loss was observed up to 250 °C, particularly due to evaporation of free, bound, and absorbed water. Afterwards, slight losses were also noted, mainly due to the decomposition of some hydrated products at temperature 400–500 °C, such as calcium sulfoaluminate and C–S–H; gehlenites, such as hydrate (C_2_ASH_8_); and dehydration of CH, as explained by Morsy et al. [140], which is also clear from the XRD patterns shown in Figure 10.

#### 3.3.3. Other Durability Properties

**Figure 11:**
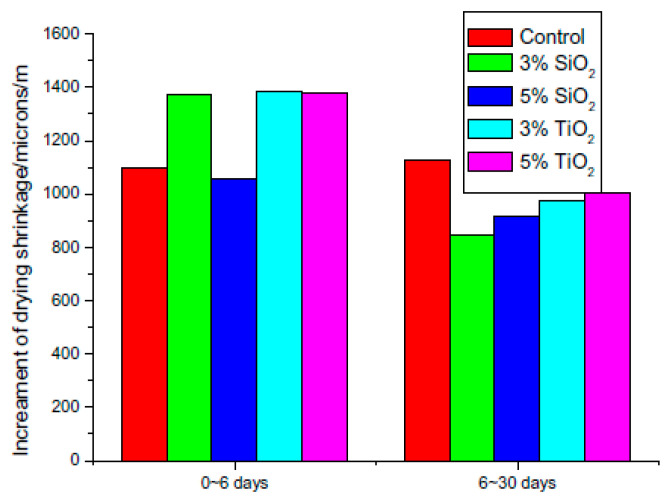
Effects of nano-SiO_2_ and nano-TiO_2_ on the drying shrinkage of cement pastes (taken with permission from Zhang et al. [21]).

Losses of strength due to sulfate attack and shrinkage strain and losses of mass due to the freeze–thaw cycle affect the durability properties, which are also greatly affected by the incorporation of nanomaterials. As highlighted in Table 3, the inclusion of nano-silica imparts durability and strength to cement mortar by filling the pores within the matrix, which reduces the mass loss due to freeze-thaw cycles and loss of strength due to sulfate attack [47,81]. As shown in Figure 11, the added nanomaterials, such as nano-titanium oxide and nano-carbon, physically fill the empty spaces, thereby decreasing the overall shrinkage strain of the cement mortar compared to the control sample [21,122].

It is known that the drying shrinkage of hardened cement mortar depends upon the stiffness and the capillary stress exerted by the concave meniscus. As per the Laplace equation, the capillary stress is proportional to the reciprocal of the radius of the curved meniscus. The lower shrinkage value of the more compact sample could be due to the higher stiffness of the base material [21].

### 3.4. Microstructural Characterization

**Figure 12:**
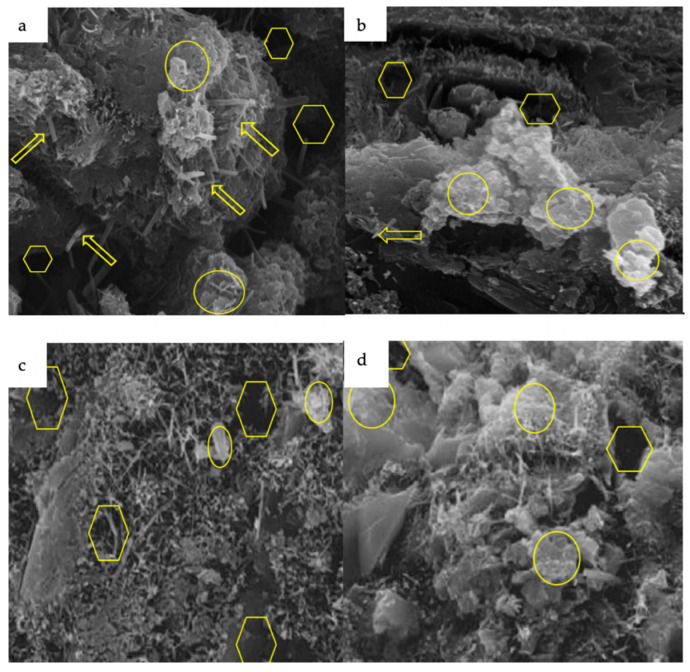
SEM images of: (**a**) control mortar sample; (**b**) nano-silica added to mortar (taken with permission from Jo et al. [94,107]); (**c**) control mortar sample; (**d**) nano-alumina added to mortar [105,117].

In the absence of nanomaterials, the mortar matrix has a dispersed structure and the voids (as shown by yellow ovals) and needle-like ettringites (as shown by yellow arrows) are more prominent than in the treated sample (Figure 12). However, the presence of nanomaterials causes the mortar to have a dense structure by reducing the number of voids and ettringites, as they can promote the formation of C–S–H gel through an accelerated hydration process [30,80,147]. However, the addition of nanomaterials accelerates the hydration process and enhances the formation of C–S–H gel (as marked by yellow hexagons), which makes the microstructure denser, thereby increasing the strength and durability of the treated sample. However, non-uniform dispersion or excessive addition of nanomaterials (beyond an optimum dosage) leads to agglomeration and formation of weak bonds in the mortar sample [77,148,149,150]. Ling et al. [13] observed that the inclusion of nano-silica in the mortar reduces the pores, concentrates the matrix, and enhances the strength of cementitious composites by improving the adhesion of the hydration products with the PVA fibers. Adding nano-titanium oxide to the mortar improves the microstructural and mechanical properties, such as compressive and flexural characteristics of composites, by consuming portlandite crystals and increasing the amount of C–S–H gel [106]. Tamimi et al. [65] showed that due to the interactions of the carboxylic groups present on the surfaces of CNTs and C–S–H, the flexural strength is increased. The interactions lead to strong covalent bonds at the interfaces between the CNT reinforcement and the matrix of cementitious compounds that bridge the microcracks.

**Figure 13:**
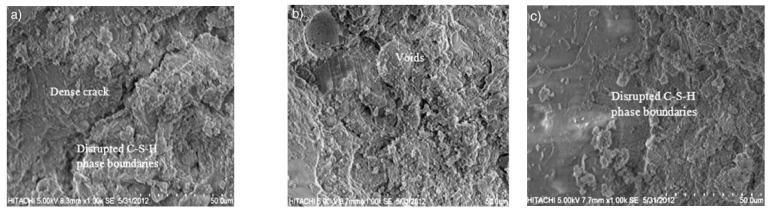
SEM images of: (**a**) control sample (0% nano-alumina); (**b**) 1% nano-alumina; (**c**) 2% nano-alumina (taken with permission from [47,48]).

Furthermore, [48] indicated that at 400 °C the dense cracks in control samples were transformed into voids and hairline cracks around the boundaries with the increase in the nano-alumina content. The decomposition of CH and C–S–H due to the release of water from hydration products may have been the reason for hairline cracks around the perimeter in specimens treated with nano-alumina. This phenomenon is apparent in SEM images (Figure 13).

## 4. Conclusions and Future Outlook

The outcomes of the literature reviewed are summarized below to indicate the impacts of the inclusion of nanomaterials in cement mortar, the properties of which vary with the type of nanomaterial used:

1. The water demand is increased for the standard consistency and the setting time is shortened with the use of nano-silica, nano-clay, nano-alumina, or nano-titanium oxide;
2. The higher consumption of mixing water leads to reduction of the workability of the cement mortar for nano-silica and nano-zinc oxide;
3. The compressive strength of the cement mortar increases and reaches a maximum value at the optimum dosage of 1.25% for nano-silica, however this varies for different cementitious nanomaterials. For nano-silica and nano-alumina, non-uniform dispersion or addition in excess of the optimum amount decreases the compressive strength due to agglomeration;
4. In addition to the increases in the elastic modulus and shear strength, the flexural and tensile strengths are also increased due to enhancement of the hydration processes for nano-silica, nano-clay, nano-alumina, nano-titanium oxide, and CNTs. It has been shown that the flexure strength decreases beyond optimum dosages (0.5%) for nano-titania, while tensile strength decreases beyond optimum dosages (1%) for nano-alumina;
5. As the durability is enhanced, the chloride permeability is reduced with the inclusion of nanomaterials. It has been observed that the decreases fornano-silica and nano-clay are due to reduction of hydroxyl groups (OH^−^ ions), while for nano-titanium oxide and carbon nano-tubes (CNTs) this is due to densification of the microstructure, which is a highly desirable change;
6. As the voids are filled and the microstructure becomes denser, the strength loss due to sulfate attack and mass loss due to freeze-thaw cycles are reduced, as in the case of nano-silica;
7. Microstructural pores are repaired or healed with the change of state of mortar from dispersed form to packed form, such as when using nano-alumina and nano-silica;
8. With the partial replacement using nanomaterials, the water absorption is reduced as the void spaces in the mortar matrix are filled up when using nano-silica or nano-clay;
9. The addition of nanomaterials such as carbon nanotubes (CNTs) and nano-titanium oxide greatly reduces the shrinkage strain;
10. The compressive strength and other hardened-state properties of nano-mortars are found to be enhanced even at elevated temperatures compared to control specimens (basic mortars). Most of the free water is evaporated at 250 °C and the decomposition of some hydrated products starts from around 400 °C with the increase in nano-alumina content.

The following are recommended as future directions of study based on the literature review and observations. These are applicable to both normal and elevated temperatures:

1. As different nanomaterials act differently when mixed with conventional cement mortar, it is important to conduct a cost–benefit analysis and to identify the overall impacts on the environment, such as acid attacks and CO_2_ emissions;
2. Given the morphology studies on the use of different nanomaterials, there is a need to assess the mechanical and durability properties resulting from the combination of fibers with nanomaterials, such as carbon fibers, polypropylene fibers, and more;
3. The application of nano-mortars to impact heat seepage in buildings [151] and for plastering purposes requires extensive study and comparisons with traditional options;
4. The evaluation of thermal conductivity and dielectric constant parameters is of paramount importance;
5. As different nanomaterials bring about varied physical and chemical changes when incorporated into cement mortars in terms of durability and strength properties, the combined effects of various types of nanomaterials need to be studied.

Nanomaterials could be used in cement mortar as good partial replacements or in addition to cement, with the added advantage that they may help in solving sustainability issues related with the cement industry. Once extensive research has been carried out in the particular field of the use of nanomaterials in cement mortar, large-scale applications should be feasible.

## Figures and Tables

**Table 1:** Properties of nanomaterials.

Nanomaterials *	Formula	Specific Surface Area (m^2^/g)	Particle Size (nm)	Form	Specific Gravity	Colour	Purity (%)	References
Nano-silica	SiO_2_	40–600	2–264	Colloidal solution, gel-type, and dry powder	1.12 to 2.6	White	SiO_2_ ≥ 98% to 99.9%	[5,18,27,28,29,30,31,32,33,34,35,36,37,38,39]
Nano-clay (Palygorskite)	-	-	3	-	2.29	-	-	[40]
Nano-clay (Halloysite)	-	65	30–70	Powder	-	White	-	[41]
Nano-clay (Kaolinite)	-	30	300–500	Powder	-	-	-	[42]
Nano-clay (Montmorillonite)	-	330	-	-	-	Light Cream	-	[43]
Nano-alumina	Al_2_O_3_	15–177	13–550	Dry powder	3.5–3.9	White	Al_2_O_3_ ≥ 98% to 99.9%	[44,45,46,47,48,49]
Nano-magnesium oxide	MgO	25–50	30–418.5	-	-	White	MgO ≥ 98% to 99.9%	[50,51,52,53]
Nano-zinc oxide	ZnO	18–60	25–60	Dry powder	-	White	ZnO ≥ 99% to 99.5%	[54,55]
Nano-titanium oxide	TiO_2_	20–240	15–50	Colloidal solution and dry powder	3.8	White	TiO_2_ ≥ 92% to 99.9%	[56,57,58,59,60]
Nano-carbon	C	40–320	5–80	Liquid solution, dry powder	-	Black	C ≥ 88% to 98.5%	[12,37,61,62,63,64,65]

* Cementitious: nano-silica (SiO_2_), nano-clay, nano-alumina (Al_2_O_3_), nano-magnesium oxide (MgO), nano-zinc oxide (ZnO). Non-cementitious: nano-titanium oxide (TiO_2_), nano-carbon (C).

**Table 2:** Natural and synthetic pozzolans in mortar.

Pozzolans Materials	Forms	Origin	Dosages	Remarks
Fly ash	Fine powder, spherical	Originates from the combustion of pulverized coal and is carried from the combustion chamber of the furnace by exhaust gases.	It is generally used at 5–65% by mass of cementitious materials.	Advantages: Used as an admixture, reduces crack problems, permeability, and bleeding; reduces heat of hydration; reduces CO_2_ emissions. Disadvantages: Slower strength gain, increased need for air-entraining admixtures, increase of salt scaling produced by higher proportions of fly ash [66].
Silica fume	Finely divided residue, spherical	Originates from the production of elemental silicon or ferro-silicon alloys that is carried from the furnace by exhaust gases.	It is generally used at 5–12% by mass of cementitious materials.	Advantages: Early strength, increased thermal conductivity, good adsorption performance, no agglomeration phenomenon. Disadvantages: Dry shrinkage rate high, reduced workability, temperature crack rate high [67].
Slag cement	Glassy, granular material, angular	Formed when molten, iron blast furnace slag is rapidly chilled typically by water sprays or immersion in water	It is used at 20–70% by mass of cementitious materials.	Advantages: Improved workability, increased cohesiveness, reduced water demand. Disadvantages: Poor water absorption, low mechanical resistance at first aging [68].
Metakaoline	Angular, platy	It is produced from relatively pure kaolinite clay.	It is used at 5–15% by mass of cementitious materials.	Advantages: Reduced bleeding and segregation. Disadvantages: In some cases poor workability and consistency [68].

**Table 3:** Effects of nanomaterials on consistency and setting time, compressive strength, flexural and tensile strength, and water absorption values.

Authors	Nanomaterials Used	Aggregates or Mineral Admixtures	Superplasticizers	Observations
Effects of Nanomaterials on Consistency and Setting Times of Cement Paste.				
[103]	Nano-silica (15 nm) - partial replacement of 1, 2, 4, and 6 wt% of cement.	-	-	- The water for standard consistency increased from 26.5% to 38.7% upon addition of 6% by wt of nano-silica. - The initial and final setting times decreased from 190 to 100 min and 345 to 250 min, respectively, upon addition of 6% by wt of nano-silica.
[103]	Nano-silica (15 nm) - addition of 0.5, 1, 2, and 3 wt% of cement.	Nano-clay (0.5%, 1%, 2%, and 3 wt% against 0.5%, 1%, 2%, and 3% nano-silica, respectively).	-	- The water for standard consistency increased considerably with the increase of combined addition of nano-silica and nano-clay. - The initial and final setting times decreased from 190 to 90 min and 345 to 240 min upon addition of 3% by wt of nano-silica and nano-clay, respectively.
[105]	Nano-silica - addition of 1, 2, and 3 wt% of cement.	Fly ash (20 wt% of binder).	-	- The water for standard consistency increased by 2–3% upon addition of nano-silica. - The setting times decreased considerably upon addition of nano-silica.
[86]	Nano-silica (15 nm) - partial replacement of 1, 1.5, 2, and 2.5 wt% of cement.	-	Polycarboxylic-acid-based superplasticizer (SP) (2 wt% of the binder).	- The initial and final setting times decreased significantly upon addition of 2.5% by wt of nano-silica.
[41]	Nano-clay - substitute levels of 1, 2, 3, 4, 5, and 6 wt% of high-volume slag cement.	-	-	- The inclusion of 6% increased the water/cement ratio from 25% to 26.7% to achieve standard consistency. However, both the initial and final setting times decreased upon addition of nano-silica up to 6% by wt.
[101]	Nano-clay - replacement levels of 1, 2, 4, 6, and 8 wt% of cement.	-	-	- The water requirements for standard consistency increased from 26.5% to 29.4% with the increase of nano-clay up to 8%. - The initial and final setting times decreased from 190 to 145 min and 345 to 270 min upon addition of 8% by wt of nano-clay, respectively.
[46]	Nano-alumina - replacement levels of 1, 2, 4, and 6 wt% of cement	.-	-	- The water for standard consistency increased from 26.5% to 36% with the increase of nano-alumina up to 6%. - The initial and final setting times decreased from 190 to 65 min and 345 to 230 min with the addition of 6% by wt of nano-alumina, respectively.
[100]	Nano-titanium oxide - replacement levels of 5, 7, and 10 wt% of cement.	Micro-silica (14.25%, 13.95%, and 13.5 wt% against each 5%, 7%, and 10% nano-TiO_2_, respectively).	SP (0.4 wt% of the solid mix).	- The water for standard consistency increased significantly upon addition of 5% of nano-TiO_2_. However, the setting times decrease upon addition of nano-silica.
[106]	Nano-titanium oxide - partial replacement levels of 1, 3, and 5 wt% of cement.	-	-	- The initial and final setting times decreased by 23.3% and 12.7% upon addition of 5% by wt of nano-TiO_2_, respectively.
Effect of Nanomaterials on Compressive Strength (CS) of mortar.				
[3]	Nano-silica (20–30 nm) - 1, 2, 3, and 4 wt% of cement.	Natural zeolite (5%, 10%, 15%, and 20% by weight of cement).	Polycarboxylate-based SP (1.1 wt% of binder).	- Addition of 3% nano-silica combined with 15% zeolite caused significant increases at 28 and 90 days of CS value of 30% and 23.9% with respect to the control mix, respectively.
[8]	Nano-silica (40 nm) - replaced by 2, 4, 6, 8, 10, 12, and 14 wt% of binder.	Fly ash + nano-silica (ratio 3:7).	Polycarboxylate-ether-based SP.	- Increases by 21.15 and 21.45 MPa after 28 and 90 days of curing, respectively, when compared to the control sample due to incorporation of 10% nano-silica.
[9]	Nano-silica (NS) - 3 wt% of cement.	Blast furnace slag (BFS) (10%, 20%, 30%, and 40 wt%).	SP (0% and 2.5% for 0% and 3 wt% of nano-silica added to mortar, respectively).	- Improvement of the CS from 50.73 to 59.42 MPa at 28 days - Optimum was 3% NS plus 30% BFS.
[11]	Nano-silica (40 nm) - inclusion levels of 1, 2, 3, and 4 wt% of binder.	Silica fume (4 wt% partial replacement of cement).	Polycarboxylate-based SP (0.13, 0.13, 0.26, 0.42, and 0.56 wt% for 0%, 1%, 2%, 3%, and 4 wt% of nano-silica added to mortar, respectively).	- The 28-day CS increased by 4.5 MPa compared to untreated sample due to addition of 2 wt% nano-silica.
[12]	Superhydrophobic nano-silica - replacement levels of 1, 2, and 4 wt%.	-	Polycarboxylate water-reducing admixture (0.3, 0.54, 0.78, and 1.26 wt% for 0%, 1%, 2%, and 7 wt% of nano-silica added to mortar, respectively).	- Addition of 4 wt% of nano-silica led to great enhancement of CS value of mortar.
[85]	Colloidal nano-silica (20 nm) - replaced by 0.5, 1, 1.5, and 2 wt% of cement.	-	-	- Increase of 11% after 28 days due to replacement with 2% nano-silica when compared to control sample.
[60]	Nano-silica (15 nm) - use of 0.5–1.5 wt%.	Unground palm oil fly ash (UPOFA) (10%, 20%, and 30 wt% against each 0.5%, 1%, and 1.5% nano-silica, respectively).	SP (1.4%, 1.5%, 1.9%, and 2.5% for 0%, 10%, 20%, and 30 wt% of UPOFA, respectively).	- Increase of the CS from 67.3 to 75 MPa and from 88.5 to 90.5 MPa at 28 and 90 days, respectively. - Optimum was 0.5% NS plus 10% UPOFA.
[107]	Nano-silica (40 nm) - use of 3%, 6%, 10%, and 12 wt%.	-	SP (1.2%, 1.8%, 2.4%, 2.9%, and 3.3 wt% for 0%, 3%, 6%, 10%, and 12 wt% of nano-silica, respectively).	- Increase of the CS from 18.3 to 50.7 MPa and from 25.6 to 68.8 MPa at 7 and 28 days, respectively. - Optimum level of 12% nano-silica.
[13]	Nano-silica (NS) (30 nm) - addition of 2 wt% of cement.	Polyvinyl alcohol (PVA) fiber (0%, 0.3%, 0.6%, 0.9%, 1.2%, and 1.5% by volume of composite) Fly ash addition by cement ratio (7:13).	High-range water-reducing admixture (0.45 wt% of cement).	- The highest CS values for 2% NS and 0.6% PVA composite soil increased by 1.03 times the strength of the untreated specimen.
[108]	Nano-silica (25 nm) - use of 2 wt%.	High-volume fly ash (HVFA) (38%, 48%, 58%, and 70 wt%).	-	- Improvement of the CS from 35 to 37 MPA in 28 days. - Optimum was 2% NS plus 38% HVFA.
[40]	Nano-clay (3 nm) - addition levels of 0.5% and 2.5%.	-	SP (varied between 0.6% and 3% by wt of the binder ).	- Huge enhancement of CS value observed upon addition of 0.5% dosage of nano-clay with SP content of 3% by wt of the binder.
[109]	Nano-alumina - replacement levels of 1%, 3%, and 5%.	-	-	- Maximum value of CS achieved at 5%, which was approximately 1.5 times higher than that of the controlled sample.
[92]	Nano-magnesium oxide (100 nm) - inclusion levels of 2.5%, 5%, and 7.5%.	-	Naphthalene-sulfonate-based SP (0.5 wt% of the cement).	- Improved the CS value from 74.7 to 80.9 MPa due to addition of 7.5 % MgO after 28 days of treatment.
[54]	Nano-zinc oxide (60 nm) - substitution levels of 1%, 3%, and 5%.	-	SP (1 wt% of the cement).	- Increased the CS value from 41.12 to 50.60 MPa after 28 days of treatment. - Optimum dosage of 3% by wt of the binder.
[10]	Nano-titanium oxide (20 nm) - replacement levels of 1%, 3%, and 5%.	Rice husk ash (RHA) (5%, 10%, and 15 wt% against each 1%, 3% and 5% nano-TiO_2_, respectively).	Polycarboxylate-ether-based SP (varied between 0.6 and 1.2% by wt of the binder).	- Great enhancement of CS value was observed due to addition of 5% nano-TiO_2_ and 10% RHA.
[94]	Nano-titanium oxide (20–30 nm) - substitute levels of 0.1%, 0.2%, 0.5%, and 1%.	-	-	- Incorporation of 0.2% nano-TiO_2_ resulted in maximum CS of 57.92 MPa as compared to controlled sample of 55.91 MPa.
[110]	CNT (8–15 nm) - added 0.05%, 0.1%, and 0.15 wt% of cement.	-	SP (0.3%, 0.45%, and 0.6 wt% for 0.05%, 0.1%, and 0.15 wt% of CNT, respectively).	- Addition of 0.1% of CNT resulted in an increase from 38.5 to 52 MPa and from 47.5 to 57.5 MPa after 28 and 90 days of curing with respect to untreated sample, respectively.
[111]	CNT (3–8 nm) - 0.005%, 0.02%, 0.05%, and 0.1 wt% of cement.	Nano-metakaolin (NMK) (6 wt%).	Dispersant solution.	- Noteworthy increase in UCS value was observed upon addition of 6% NMK and 0.02% CNT as compared to untreated sample.
[62]	CNT (30–80 nm) - 0.01%, 0.03%, 0.05%, and 0.07 wt%.	Nano-silica (NS) (1 wt%).	Polycarboxylate-based SP (varied from 0.5 to 1 wt%).	- Sample with 1% NS and 0.01% CNT showed significant CS value. The increase was 62% compared to pure ground values after 28 days of curing.
Effects of Nanomaterials on Flexural strength (FS) and Tensile Strength (TS) of Mortar.				
[82]	Nano-silica sol (25–35 nm) - use of 0.5, 1, 1.5, 2, and 3 wt% of cement.	-	Poly-carboxylic acid water-reducing agent (1 wt% of binder).	- Increase of the FS from 9 to 10.7 MPa in 7 days. - Optimum was 3% nano-silica.
[89]	Nano-silica sol (30–100 nm) - addition of 2, 6, and 10 wt% of cement.	-	-	- Increase of the TS from 2.2 to 2.65 MPa in 28 days. - Optimum was 2% nano-silica.
[112]	Nano-silica (30 nm) - substitute levels of 1, 2, 3, and 4 wt% of cement.	Silica fume (SF) (16.67 wt% of cement).	Polycarboxylate-based SP (3.33 wt% of binder).	- The 28-day FS increased from 28.2 to 37.3 MPa due to addition of 4 wt% nano-silica.
[43]	Nano-clay - substitute levels of 1%, 3%, 5%, 7%, and 10%.	-	-	- Due to addition of 10% nano-clay, the FS values increased by 76.6%, 67.2%, and 65.4% after 3, 7, and 28 days of curing, respectively.
[79]	Nano-clay - replacement levels of 0.5%, 1%, and 2 wt%.	-	-	- Due to addition of nano-clay, TS increased from 2.8 to 3.1 MPa and from 3.2 to 3.5 MPa after 7 and 28 days of treatment, respectively.
[10]	Nano-alumina (20 nm) - substitute levels of 1%, 2%, and 3%.	Rice husk ash (RHA) (10%, 20%, and 30 wt% against each 1%, 2%, and 3% nano-alumina, respectively).	Polycarboxylate-based SP (varied between 0.2% and 1% by wt of the binder).	- The FS value reached 9.06 MPa when the amount of nano-alumina used was 3% and RHA was 10% compared to 6.76 MPa for the controlled sample after 28 days of curing.
[113]	Nano-alumina (15 nm) - substitute levels of 0.5, 1, 1.5, and 2 wt%.	-	-	- TS increased with nano-alumina up to 1% replacement and then decreased, although the value against 2% was still higher than the control mix.
[114]	Nano-titanium oxide (21 nm) - replacement levels of 0.25%, 0.5%, and 1% wt.	-	-	- The FS value increased significantly with increase in nano-TiO_2_ content from 0.25% to 0.5%, then beyond 0.5% the strength decreased.
[95]	CNT (20–45 nm) - (0.08%, 0.1%, 0.3%, and 0.5 wt%).	-	SP (0.008 wt%).	- The FS value increased from 5.9 to 11.1 MPa on inclusion of 0.1% of CNT after 28 days, leading to an increase of 88.1% with respect to untreated sample.
[115]	CNT (50 nm) - addition levels of 0.5 and 1 wt% of cement.	Silica fume (SF) (10 wt% of partial replacement of cement).	-	- Significant improvement in FS value was observed on inclusion of 0.5% of CNT and 10% of SF.
[116]	CNT (6–25 nm) - addition of 0.05% and 0.1 wt% of cement.	-	-	- Addition of 0.05 wt% CNT increased the TS by 20.58% as compared to controlled sample in 28 days.
[12]	CNT - use of 0.01–0.06 wt% of cement.	Hemp fibers (HF) (2.1% volume).	-	- Maximum FS value was observed on addition of 0.01% of CNT and 2.1% HF with respect to that of controlled sample.
Effects of Nanomaterials on Water Absorption (WA) of Mortar.				
[27]	Nano-silica (8–20 nm) - addition of 1.5 and 3 wt% of cement.	-	-	- Increase in amount of nano-silica up to 3% caused decreases WA (%) in mortar.
[79]	Nano-clay - substitute levels of 0.5, 1, and 2 wt% of binder.	-	-	- Inclusion of 0.5% nano-clay decreased the WA (%)compared to the control sample and then increased with increase in nano-clay content up to 2%.
[117]	Nano-alumina - replacement levels of 1, 3, and 5 wt% of cement.	-	-	- Decreased the WA (%) from 8.37% to 7.8% in 28 days. - Optimum dosage was 1 wt%.
[10]	Nano-alumina (20 nm) - substitute levels of 1%, 2%, and 3%.	RHA (10%, 20%, and 30 wt% against each substitute level).	Polycarboxylate-based SP (varied between 0.2% and 1% by wt of the binder ).	- By adding 3% nano-silica and 20% RHA, the WA (%) was 0.8 times lower than untreated specimen after 28 days of curing.
[118]	Nano-titanium oxide (15 nm) - replacement levels of 1, 3, and 5 wt% of cement.	-	Polycarboxylate-based SP (varied between 0.75 and 0.85% by wt of the binder ).	- The WA (%) of the treated sample decreased rapidly at 5% in comparison to the untreated sample.

**Table 4:** Effects of nanomaterials on slump values, mechanical properties, chloride penetration values, and other durability properties of mortar.

Authors	Nanomaterials Used	Superplasticizers	Observations
Effects of nanomaterials on Slump Values of Mortar.			
[59]	Nano-silica (20 nm) - partial replacement of 0.75, 1.5, and 3 wt% of cement.	SP (1.5 wt% of cement).	- The water for standard consistency increased from 26.5% to 38.7% upon addition of 6% by wt of nano-silica. - The initial and final setting times decreased from 190 to 100 min and 345 to 250 min upon addition of 6% by wt of nano-silica, respectively.
[109]	Nano-alumina - replacement levels of 1%, 3%, and 5%.	-	- The initial and final setting times decreased significantly upon addition of 2.5% by wt of nano-silica.
[55]	Nano-zinc oxide (25 nm) - addition of 1%, 3%, and 5 wt%.	-	- The water for standard consistency increased by 2–3% upon addition of nano-silica. - The setting times decreased considerably upon addition of nano-silica.
[119]	Nano-zinc oxide (12 nm) - partial replacement levels of 0.05%, 0.1%, and 0.2 wt%.	-	- The water for standard consistency increased considerably with the increase of the combined addition of nano-silica and nano-clay. - The initial and final setting times decreased from 190 to 90 min and 345 to 240 min upon addition of 3% by wt of nano-silica and nano-clay, respectively.
Effects of nanomaterials on other mechanical properties of mortar.			
[32]	Nano-silica (36 nm) - substitute levels used were 0.5%, 1%, 1.5%, 2%, 3%, and 4 wt%.	-	Increase the shear stress. Optimum (%) = 1.5%
[48]	Nano-alumina (15 nm) - replacement levels used were 1%, 2%, and 3 wt%.	Napthalene-sulfonate-based SP (5% by wt of the total composite).	Increase the elasticity modulus. Optimum (%) = 1%
[95]	CNT (20–45 nm) - use of 0.08%, 0.1%, 0.3%, and 0.5 wt%.	SP (0.008% by wt of the binder).	Increase the elasticity modulus. Optimum (%) = 0.1%
Effect of nanomaterials on chloride penetration of mortar.			
[81]	Nano-silica (5–20 nm) - addition of 1 and 2 wt% of cement.	Polycarboxylate-based SP.	- The total charge passed decreased from 5254 to 4761 coulombs, thereby enhancing the chloride resistance. - The optimum dosage was 1 wt%.
[120]	Nano-clay - addition levels of 1, 2, 3, 5 m and 7 wt% of cement.	-	- Minimum total charge passed was obtained when 2% of nano-clay was added to the specimen.
[118]	Nano-alumina (15 nm) - replacement levels of 1, 3, and 5 wt% of cement.	Polycarboxylate-based SP (varied between 0.75 and 0.85% by wt of the binder).	- Slight decrease in total charge passed was obtained when 3% dosage of CNT was partially replaced with cement.
	Nano-titanium oxide (15 nm) - replacement levels of 1, 3, and 5 wt% of cement.		- Huge decrease in total charge passed was obtained when 5% dosage of nano-titanium oxide was partially replaced with cement.
[121]	CNT (20–40 nm) - addition of 0.2, 0.4, 0.6, and 0.8 wt% of cement.	Polycarboxylate-based SP.	- The lowest total charge passed was obtained when 0.8% dosage of CNT was partially replaced with cement, leading to an increase in chloride resistance.
Effects of nanomaterials on other durability properties of mortar.			
[47]	Nano-silica (12 nm) - 5 wt% of cement.	Polycarboxylate-based SP (1.2% by wt of cement).	Reduction in mass loss due to freeze–thaw cycle. Optimum (%) = 5%
[81]	Nano-silica (5–20 nm) - addition of 1 and 2 wt% of cement.	SP (varied between 1.2% and 2.5%).	Reduction in loss of strength due to sulfate attack. Optimum (%) = 2%
[122]	CNT (15 nm) - addition of 0.1 and 0.3 wt% of cement.	SP (2 wt% of cement).	Decrease the shrinkage strain. Optimum (%) = 0.3%
[21]	Nano-titanium oxide (25 nm) - replacement levels of 1, 3, and 5 wt% of cement.	SP (varied between 1% and 2.7% to maintain slump flow of 165 mm).	Decrease the shrinkage strain. Optimum (%) = 5%
